# EXAMINING CUMULATIVE INEQUALITY IN THE ASSOCIATION BETWEEN CHILDHOOD SES AND BMI FROM MIDLIFE TO OLD AGE

**DOI:** 10.1093/geroni/igz038.1274

**Published:** 2019-11-08

**Authors:** Chioun Lee, Chioun Lee, Soojin Park

**Affiliations:** University of California-Riverside, Riverside, California, United States

## Abstract

Socioeconomic status (SES) is among the strongest determinants of body mass index (BMI). For older populations, selection bias is a large barrier to assessing cumulative disadvantages. We investigated the extent to which childhood SES affects BMI from midlife to old age and gender differences in the association. Data come from Midlife in the U.S. We used latent growth models to estimate BMI trajectory over a period of 20 years and examined results under different missing data patterns. Compared to individuals from higher childhood SES, those from lower childhood SES have higher BMI in midlife and experience a faster increase in BMI between midlife and old age. The observed associations remain significant even after controlling for midlife SES. After addressing nonrandom selection, the gap in BMI between high and low childhood SES widens from midlife to old age for women. The findings provide new evidence of cumulative inequality among older adults.

